# Membrane potential dynamics unveil the promise of bioelectrical antimicrobial susceptibility Testing (BeAST) for anti-fungal screening

**DOI:** 10.1128/mbio.01302-24

**Published:** 2024-07-23

**Authors:** Tailise Carolina de Souza-Guerreiro, Letícia Huan Bacellar, Thyerre Santana da Costa, Conor L. A. Edwards, Ljubica Tasic, Munehiro Asally

**Affiliations:** 1 School of Life Sciences, University of Warwick, Coventry, United Kingdom; 2 Warwick Integrative Synthetic Biology Centre, University of Warwick, Coventry, United Kingdom; 3 Institute of Chemistry, Biological Chemistry Laboratory, University of Campinas, Campinas, Brazil; 4 Cytecom Ltd., Coventry, United Kingdom; Universidade de Sao Paulo, Sao Paulo, Brazil

**Keywords:** microbial electrophysiology, membrane potential, bioelectricity, live-cell detection, bioelectrical engineering, anti-fungal susceptibility testing

## Abstract

Membrane potential is a useful marker for antimicrobial susceptibility testing (AST) due to its fundamental roles in cell function. However, the difficulties associated with measuring the membrane potential in microbes restrict its broad application. In this study, we present bioelectrical AST (BeAST) using the model fungus *Saccharomyces cerevisiae*. Using fluorescent indicators [DiBAC4(3), ThT, and TMRM], we measured plasma and mitochondrial membrane-potential dynamics upon electric stimulation. We find that a 2.5 second electric stimulation induces hyperpolarization of plasma membrane lasting 20 minutes in vital *S. cerevisiae*, but depolarization in inhibited cells. The numerical simulation of FitzHugh-Nagumo model successfully recapitulates vitality-dependent dynamics. The model also suggests that the magnitude of plasma-membrane potential dynamics (PMD) correlates with the degree of inhibition. To test this prediction and to examine if BeAST can be used for assessing novel anti-fungal compounds, we treat cells with biogenic silver nanoparticles (bioAgNPs) synthesized using orange fruit flavonoids and Fusarium oxysporum. Comparing BeAST with optical density assay alongside various stressors, we show that PMD correlates with the magnitude of growth inhibitions. These results suggest that BeAST holds promise for screening anti-fungal compounds, offering a valuable approach to tackling antimicrobial resistance.

**IMPORTANCE:**

Rapid assessment of the efficacy of antimicrobials is important for optimizing treatments, avoiding misuse and facilitating the screening of new antimicrobials. The need for rapid antimicrobial susceptibility testing (AST) is growing with the rise of antimicrobial resistance. Here, we present bioelectrical AST (BeAST). Combining time-lapse microscopy and mathematical modeling, we show that electrically induced membrane potential dynamics of yeast cells correspond to the strength of growth inhibition. Furthermore, we demonstrate the utility of BeAST for assessing antimicrobial activities of novel compounds using biogenic silver nanoparticles.

## INTRODUCTION

Living cells maintain their internal ionic conditions to be distinct from their external environment. For example, potassium is highly accumulated inside cells in almost all species (1). The ionic difference across the membrane gives rise to an electrical potential gradient, known as membrane potential (2). The membrane potential is a component of the ion motive force that provides a source of free energy for cells to do various chemical and mechanical work, such as membrane transport and ATP synthesis (3–5), and thereby fundamental to cellular homeostasis and bioenergetics. Consequently, membrane potential is used as an indicator for proliferative capacity (cell vitality) (6), which holds paramount importance across a wide range of biological and biomedical applications, including the diagnosis of microbial infections, detection of proliferative cells, and examinations of synthetic-biology genetic circuits (7–10). Interestingly, recent studies also suggest that the resting plasma membrane potential may be related to cancer progression (11, 12), indicating a fundamental link between the plasma membrane potential and proliferative capacity.

The plasma membrane potential can be influenced by an externally applied electric field (EEF), as described by the Schwan equation: Δψ=1.5rEcos⁡θ, where r is the radius of the cell, E is the field strength, and θ is the angle to the electric field (13). The change in membrane potential (Δψ) can then trigger the opening of voltage-gated ion channels (14). Since ion flux through channels follows the electrochemical gradient, the electrically induced membrane potential dynamics could be expected to be impacted by cell vitality (the capacity of cells to proliferate). In alignment with this idea, we previously showed proliferative-capacity-dependent dynamics in bacteria (15, 16). In bacteria, the plasma membrane is the primary site of ATP synthesis, which gives a possible explanation why bioenergetic status can be measured by the electrically induced membrane potential dynamics. On the contrary, in eukaryotes, ATP synthesis mainly occurs across the inner mitochondrial membrane, not at the plasma membrane. It is, therefore, unclear whether bioenergetic state of a eukaryotic cell alters the plasma-membrane potential dynamics (PMD).

Recently, a rise of fungal threats to human and ecosystem health and increased cases of antimicrobial resistance (AMR) has been observed (17). The current protocol for anti-fungal susceptibility testing is labor-intensive and time-consuming, posing a practical barrier to addressing AMR. Electrically induced membrane potential dynamics of fungi cells could be a useful approach for antimicrobial susceptibility testing (AST) to evaluate novel and existing anti-fungal compounds and to optimize resistance surveillance. However, whether plasma membrane potential response to electric stimuli may correspond to eukaryotic cell vitality is unknown.

In this study, we investigate the electrically induced membrane potential dynamics and its application for AST using the model organism *S. cerevisiae*. Our results suggest that the PMD shows hyperpolarization in proliferative cells, but not in inhibited cells. Furthermore, mildly stressed cells showed an attenuated PMD response. Intriguingly, the dynamics correlate with the cellular growth rates, suggesting that electrically induced membrane potential dynamics can be used for bioelectrical AST (BeAST) to estimate the strength of growth inhibition.

## RESULTS

**Fig 1:**
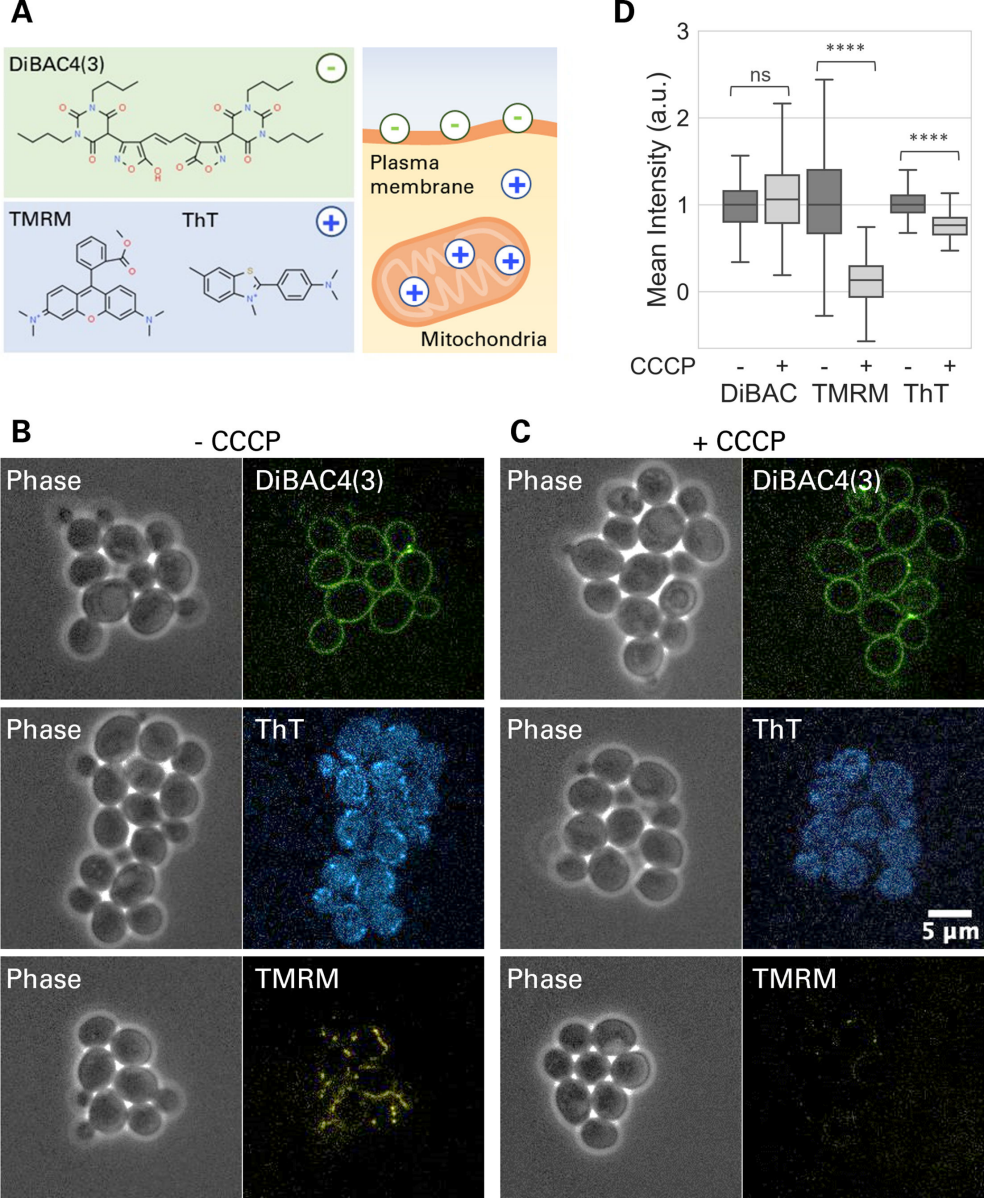
Plasma and mitochondrial membrane potential of *S. cerevisiae were monitored by fluorescece microscopy*. (**A**) Illustrative diagram depicting the chemical structures of DiBAC4(3), TMRM, and ThT. DiBAC4(3) is negatively charged and report on the plasma membrane potential. TMRM is positively charged and accumulates in the mitochondria. ThT is also positively charged and has been used to measure membrane potential. (**B, C**) Microscopy images of *S. cerevisiae* cells shown in phase contrast (left) and fluorescence (right), (**B**) without carbonyl cyanide chloro phenylhydrazone (CCCP) and (**C**) with 10 µM CCCP. (**D**) Box plot (median, 25th percentile, 75th percentile and range) showing the ratio change in the cellular fluorescence signals without and with CCCP treatment. Fluorescence intensities per cell were measured for individual cells. DiBAC4(3) (*n* > 60 cells, *P* = 0.34), TMRM (*n* > 300 cells, *P* < 0.01), and ThT (*n* > 300 cells, *P* < 0.01) from three biological repeats were analyzed.

Although the EEF only directly interacts with the plasma membrane, the mitochondrial membrane plays a more central role in cell vitality. Therefore, we investigated the electrically induced dynamics of both plasma and mitochondrial membrane potential. To measure the plasma and mitochondrial membrane potential of *S. cerevisiae* cells, we used three fluorescent membrane-potential indicators: namely, bis-(1,3-dibutylbarbituric acid) trimethine oxonol [DiBAC4(3)], tetramethylrhodamine methyl ester (TMRM), and thioflavin T (ThT) (Fig. 1A). DiBAC4(3) is an anionic dye that accumulates more in a depolarized plasma membrane; hence, its fluorescence intensity per cell increases as the plasma membrane potential becomes more positive and decreases when it becomes more negative (18). TMRM is a cationic dye that is well established as a mitochondrial membrane-potential indicator that accumulates more in negatively polarized mitochondria (19). ThT is a cationic benzothiazole dye that is most commonly used as a marker for amyloid fibrils, but it is recently used also as a membrane-potential indicator in bacteria (15, 20, 21) and also in mammalian cells (22).

To characterize the dyes in our experimental setup, we cultured *S. cerevisiae* cells on an agarose pad consisting of the yeast nitrogen base (YNB) medium and supplemented with the membrane potential indicators. As expected, the fluorescence signals of DiBAC4(3) and TMRM are localized to the plasma membrane and in the mitochondria, respectively (Fig. 1B). ThT showed mitochondrial accumulation similar to TMRM although its signal appeared more diffused in the cytoplasm than TMRM (Fig. 1B; Fig. S1).

To test if this mitochondrial accumulation of ThT depends on the mitochondrial membrane potential, we treated cells with a mild level, at 10 µM, of a proton decoupler, carbonyl cyanide chloro phenylhydrazone (CCCP) which depolarizes the mitochondrial membrane but not the plasma membrane in eukaryotes (23). As expected, treatment with 10 µM CCCP resulted in the reduction of TMRM signal, but not DiBAC4(3) signal (Fig. 1C and D). Note that this means that CCCP does not induce detectable changes in the plasma membrane potential. The fluorescence image of ThT showed a diffusive cytoplasmic pattern without a mitochondrial localization signal (Fig. 1C). This loss of mitochondrial localization of the signal suggests that ThT accumulates in the mitochondria in a membrane-potential-dependent manner. However, the diffusive signal of ThT was also visible. The quantification of the fluorescence signal confirmed that CCCP treatment only mildly reduced the ThT signal per cell (Fig. 1D). These results suggest that ThT localizes in mitochondria, but the mean ThT signal per cell is not highly sensitive to the mitochondrial membrane potential due to its diffusive cytoplasmic signal.

### The plasma membrane becomes more negative after electric shock

**Fig 2:**
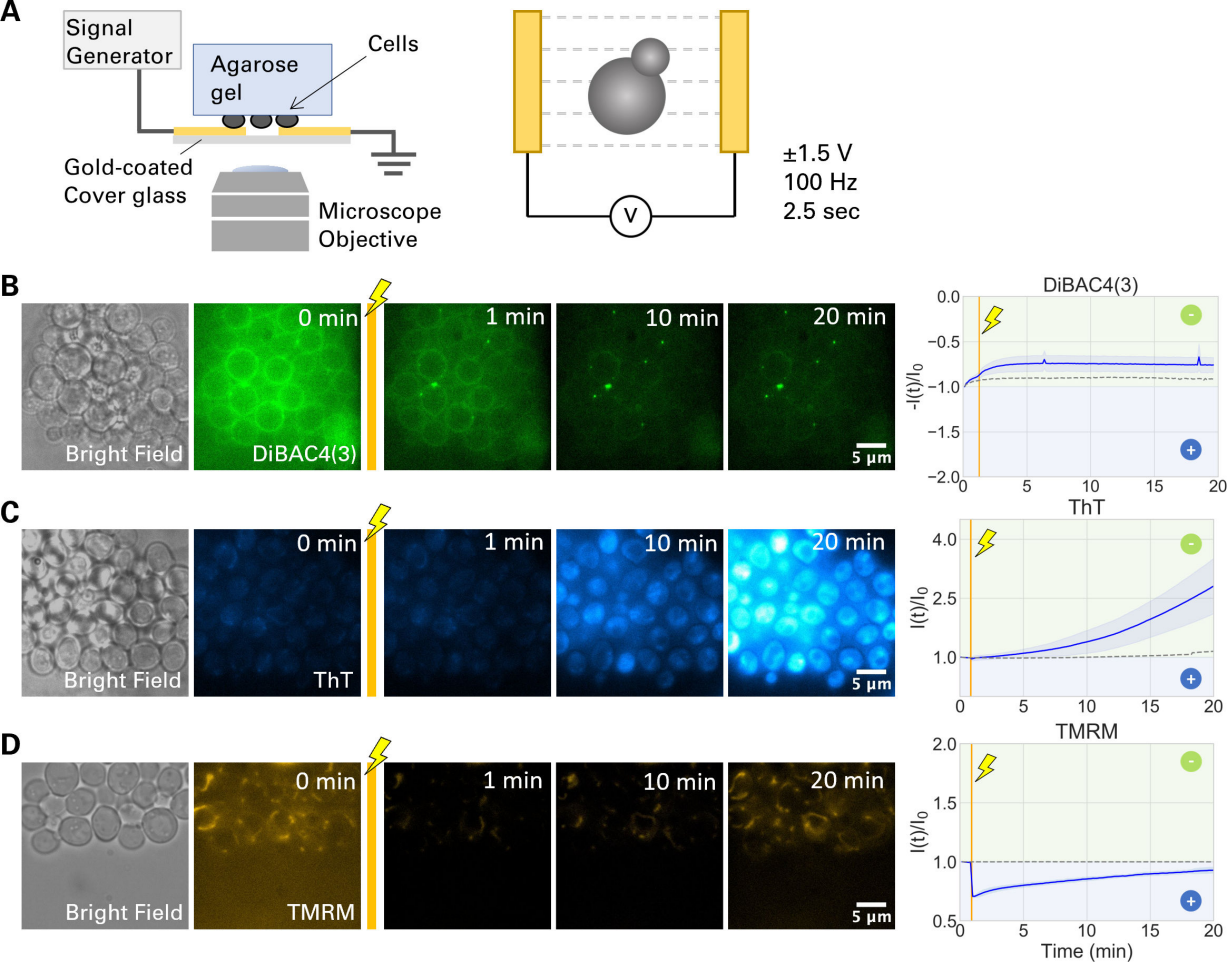
Electric stimulation induces plasma membrane hyperpolarization. (**A**) Schematic illustration of the experimental setup. Yeast cells on an agarose pad are placed facing down onto gold electrodes on a coverglass-bottom dish, connected to a signal generator, and mounted to an inverted fluorescence microscope. A ± 1.5 V, 100 Hz stimulus is applied for 2.5 s. (**B–D**) Proliferative cells exhibit a hyperpolarization in response to EEF. (**B–D**) Bright field images and film strips of fluorescence microscopy images before and after electrical stimulation. Graphs showing the fold change in fluorescence signal over time (blue solid line). Shaded blue shows standard deviation. Dashed lines are the condition without electrical stimulation. The color in the background in the graphs shows the direction of change in membrane potential: limegreen shows membrane potential going further negative, blue shows it going more positive. Gold vertical line indicates 2.5 s electrical stimulation. (**B**) DiBAC4(3) signal shows a quick and stable hyperpolarization, depicted by a decrease in fluorescence intensity. (**C**) ThT signals show a gradual hyperpolarization, with threefold increase in fluorescence intensity. (**D**) TMRM signals show an instant depolarization in response to EEF, followed by a gradual return to the initial resting membrane potential.

To examine the electrically induced plasma and mitochondrial membrane potential dynamics, YNB agarose pads carrying yeast cells were placed on a gold-coated electrode dish and used for stimulation experiment (Fig. 2A). The dynamics of fluorescence signals before and after a 2.5 s electric shock were monitored for 20 min by microscopy. The fold change in fluorescence signal over time [*I*(*t*)/*I*_0_] was used to quantify the response dynamics.

Following the 2.5 s stimulation, DiBAC4(3) signal decreased by ~25% over the period of 20 min, suggesting hyperpolarization of the plasma membrane (Fig. 2B; Movie S1). The hyperpolarization following electric stimulation was also observed with ThT, which showed ~3× rise in the signal (Fig. 2C; Movie S2). The ThT signals after electric stimulation were more diffusive in the cytoplasm, which implied less mitochondrial accumulation due to mitochondrial membrane depolarization. TMRM signal showed a rapid decline after an electric stimulation, suggesting mitochondrial depolarization (Fig. 2D; Movie S3). In the absence of electric stimuli, no significant change in the fluorescence signal was observed over the course of the 20 min time-lapse experiments (Fig. S2). The dynamics of individual cells for all three dyes are presented in Fig. S3. These results suggest that the electric stimulation hyperpolarizes the plasma membrane while it depolarizes the mitochondrial membrane.

### Cell vitality is required for electrically induced hyperpolarization

**Fig 3:**
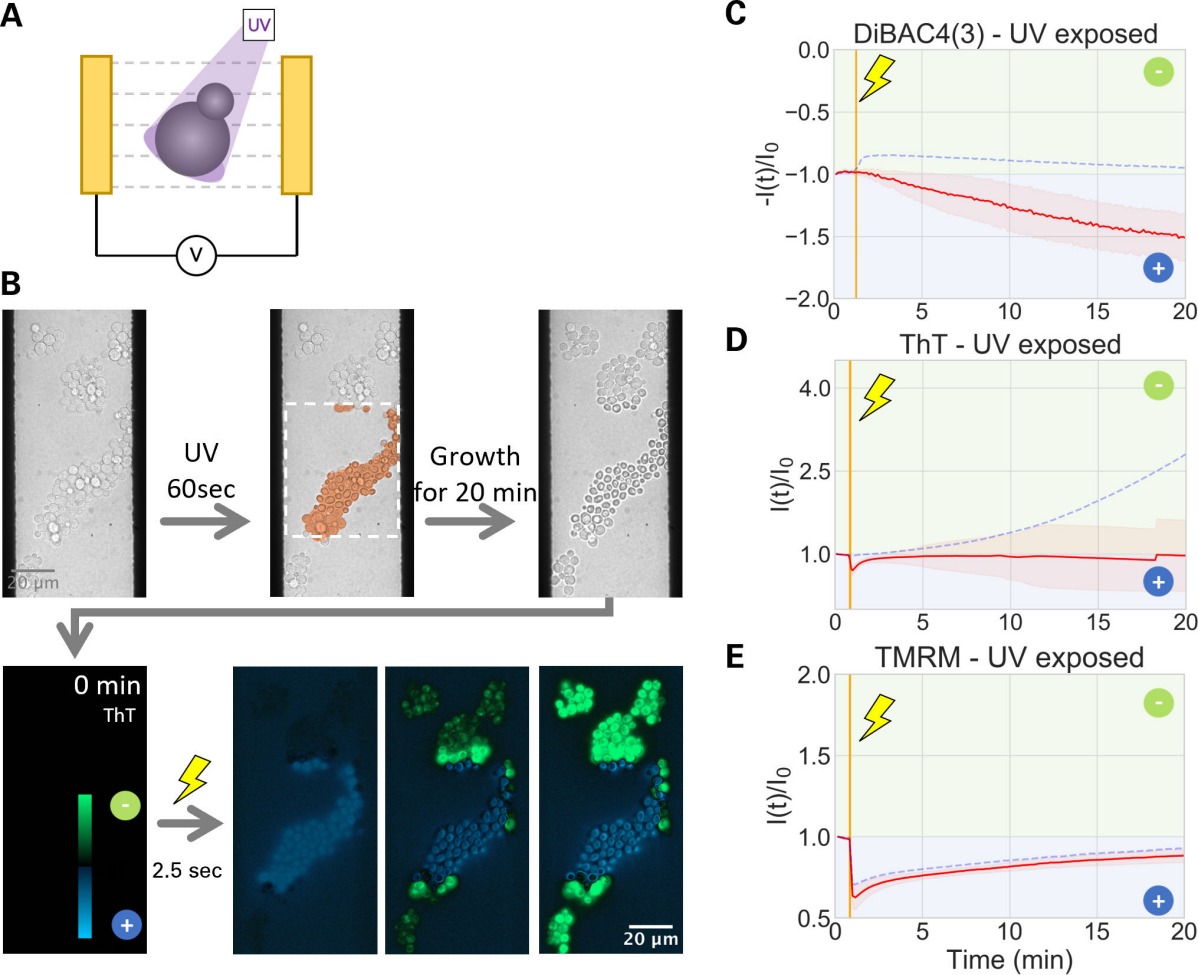
UV-irradiated cells do not produce a hyperpolarization response to EEF. (**A**) Schematic illustration of experimental design. (**B**) Flowchart diagram showing the experimental design. Bright field images show *S. cerevisiae* cells within the electrode gap. UV-violet irradiation for 60 s is applied only to the center of the field of view (highlighted by the dashed rectangle). The growth inhibition by the irradiation is verified by further incubation for 20 min and brighlight field time-lapse microscopy (see also Fig. S3 and S4). Cells are then exposed to EEF for 2.5 s. (**C**) The ratio change of ThT signal in log scale is calculated from microscopy [*I*(*t*)/*I*_0_] and shown in a green-black-blue pallete. Green shows hyperpolarization, and blue shows depolarization. The images correspond to panel B. (**D–F**) Graphs showing the fold change in fluorescence intensities of UV-V irradiated cells (solid red line) and unirradiated cells (dashed blue). Shaded red shows the standard deviation. For the negative control without electrical stimulation, see Fig. S2. The color in the background in the graphs shows the direction of change in membrane potential: limegreen shows membrane potential going further negative, and blue shows it going more positive. Gold vertical line indicates 2.5 s electrical stimulation. (**D, E**) DiBAC4(3) and ThT show a lack of hyperpolarization response for UV-V irradiate cells (solid line) but not for non-irradiate cells (dashed line). (**F**) TMRM shows an instant depolarization followed by a gradual recovery, regardless of UV-V irradiation.

To examine if the electrically induced dynamics of plasma and mitochondrial membrane potential relate to cell vitality, we used UV-violet light to inhibit cells. Specifically, the cells within the center region of the field of view were exposed to UV light for 1 min, whereas cells in other locations were not (Fig. 3A and B). This experimental design enabled us to directly compare UV-exposed and non-exposed cells within the same field of view while ensuring that they received identical electric shock.

We first confirmed by bright-field time-lapse microscopy that UV-exposed cells stopped growing (Fig. 3B; Fig. S4 and S5). The exposed cells also appeared severely damaged in brightfield images and fluorescence images (Fig. S5 and S6). With all three fluorescence dyes that we tested, fluorescence signals were brighter and diffusive in the cytoplasm (Fig. S6). Both anionic and cationic dyes increased their signals diffusively in the cytoplasm, suggesting that this rise in intensity is not specific to membrane potential, and their application as membrane potential indicators is limited with these cells. With this potential limitation in mind, UV-exposed cells were stimulated by an electric field and the fluorescence signals were monitored for another 20 min (Fig. 3). We analyzed the fold change in fluorescence intensities over time [*I*(*t*)/*I*_0_]. The response dynamics of TMRM signal was indifferent between UV-exposed and non-exposed cells and showed a transient drop in fluorescence signal (Fig. 3F; Fig. S7). This could be due to the fact that the mitochondrial membrane is insulated by the plasma membrane and would not be directly exposed to EEF.

The anionic dye DiBAC4(3) showed a ~50% rise in exposed cells (Fig. 3D; Fig. S7), which is in a clear contrast to non-exposed cells (Fig. 3D, dashed line). Intriguingly, electrically induced dynamics of ThT signal was also different between non-exposed and exposed cells. In exposed cells, ThT signal showed a slight transient decrease followed by a largely flat signal (Fig. 3E; Fig. S7), while non-exposed cells showed a gradual rise of ThT signal (Fig. 3E, dashed line). The ratio changes in ThT fluorescence are visualized in Fig. 3C. In these images, a small number of cells within the irradiated region showed ThT signal increase near an electrode (Fig. 3B; Movie S4). However, closely examining the pre-shock bright-field images, we found that these cells entered the region during the incubation period, hence, were not exposed to UV light. Putting together the dynamics of an anionic dye, DiBAC4(3), and a cationic dye, ThT, our results strongly suggest that the UV-inhibited yeast cells are unable to produce a hyperpolarization response to electric stimulation. In other words, cells need to be vital to produce an electrically induced PMD.

To further examine if stressed cells show altered response dynamics of membrane potential, we performed the assay with the cells treated with ethanol. Ethanol-treated cells showed plasma membrane depolarization upon electric stimulation (Fig. S8). These results suggest that membrane potential response to electric stimulation can be used for rapid AST, which we name as BeAST.

### The FHN mathematical model recapitulates vitality-dependent hyperpolarization

The cell-vitality-dependent hyperpolarization prompted a question about how mildly stressed, but not fully inhibited, cells react to an electric shock. In particular, we wonder if the response dynamics is gradual or binary. To explore this question, we employed the FitzHugh-Nagumo (FHN) model, a phenomenological mathematical model for neural activity (24). The FHN model is an ODE system with a cubic non-linear function for membrane potential (*V*) and a linear function for a recovery variable (*W*) which represents slow negative feedback. In this model, similar to our previous study on bacteria electrophysiology (15), vital cells accumulate high level of potassium inside the cell, which can be released upon electric shock. On the other hand, because maintaining the membrane potential is a major energetic burden (25), non-vital cells are unable to accumulate high intracellular potassium, leading to the absence of hyperpolarization.

**Fig 4:**
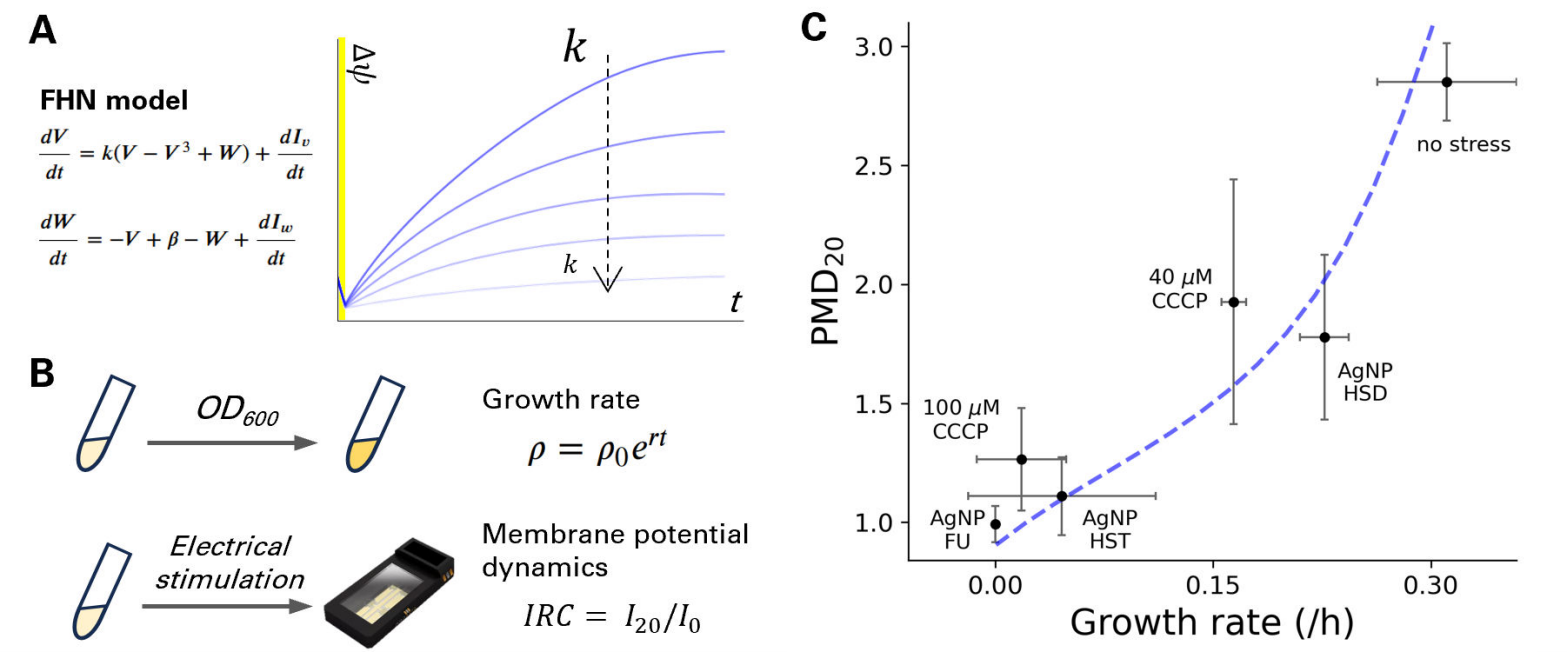
Electrically induced plasma membrane potential change correlates with growth rates. (**A**) The FHN model is used to simulate the electrically induced membrane potential dynamics. Simulations are performed with various values for k (0.9, 0.7, 0.5, 0.3, 0.1), and the changes in membrane potential (Δψ) over time are plotted. (**B**) Cells in various conditions are cultured to estimate the growth rate (r) and the PMD of electrically induced membrane potential change at 20 min (PMD_20_) using ThT. (**C**) The relationship between growth rate and electrically induced membrane potential dynamics. To calculate the growth rates, OD_600_ was measured in liquid culture. PMD_20_ was calculated from fluorescence time-lapse microscopy images. Text in the graph shows stressors. Spearman *r* value = 0.89, *P* value = 0.019. The light blue dashed line shows the FHN simulation with various *k* values. Error bars are standard deviation.

We numerically simulated the dynamics of the plasma membrane potential after an electric stimulation with varying levels of cell vitality (k). This parameter k has two effects on the model behavior. First, it shifts the equilibrium point, thus the resting membrane potential levels. Second, it determines the time scales of membrane potential dynamics, relative to the time scale of recovery variable (*W*). More specifically, with smaller k (i.e., less cell vitality), membrane potential dynamics becomes more gradual. As a result, smaller k gives rise in a weaker hyperpolarization response. This dependency on k illustrated by simulation results indicates a smaller hyperpolarization response with lower cell vitality (Fig. 4A).

### Milder stress reduces hyperpolarization level

To test this conjecture from the model, we performed plate-reader assays to measure OD_600_ for 22 h and calculated the growth rate (*r*) for the cells treated with and without stressors (Fig. 4B). We also performed BeAST with the cells treated with stress agents (Fig. 4B). ThT was used for this experiment because it showed a greater fold-change difference between UV-damaged and untreated cells than DiBAC4(3) (Fig. 3E). The intensity before and after electric shock was calculated to quantify the magnitude of plasma membrane hyperpolarization (PMD).

We used the proton decoupler CCCP as a known chemical stressor. Note that CCCP does not cause a detectable change in the plasma membrane potential (Fig. 1). Culturing cells with varying concentrations of CCCP between 0 and 100 µM, we observed a dose-dependent growth inhibition of CCCP (Fig. S9). The growth rates were reduced by ~50% and ~90% with the addition of 40 and 100 µM CCCP, respectively.

For the electrical stimulation experiment, cells were exposed to 40 and 100 µM CCCP for 1 h and used for time-lapse fluorescence microscopy. The membrane potential dynamics before and after electrical stimulation was monitored using ThT (Fig. S9) and PMD_20_ was calculated. Intriguingly, PMD_20_ was lowered by CCCP in a dose-dependent manner. Plotting PMD_20_ against the growth rate with and without CCCP, we find a correlation between growth rate and PMD_20_ (Fig. 4C). This result suggests that membrane potential dynamics can be used for assessing antifungal activities.

### BeAST can be used for examining antifungal activities of silver nanoparticles

The above findings suggest that BeAST can accelerate the assessments of novel antifungals as it can be completed in a few hours while a growth assay takes overnight. We explored this possibility using biogenic silver nanoparticles (bioAgNPs), promising alternative to antibiotics. We used the methods that can reproducibly produce stable AgNPs (26–28). The methods are also economically and ecologically beneficial as they can utilize orange wastes and do not produce toxic waste in the process. Following a few non-time-consuming steps, we obtained AgNP-HST and AgNP-HSD using orange fruit flavonoids. We also produced bioAgNP using the filamentous fungus *Fusarium oxysporum* (27). The basic properties of bioAgNPs were characterized by electron microscopy, spectroscopy, and dynamics light scattering (Fig. S13 through S15; Table S1). *S. cerevisiae* cells were incubated with AgNPs for 1 h in liquid culture and further incubated on pads for <3 h before stimulated by an electric shock. The PMD_20_ was then measured using fluorescence time-lapse microscopy images. AgNP-FU-treated cells were reminiscent of the UV-exposed cells and showed a reduced PMD_20_ (Fig. S10). Among two orange NPs, HST showed a greater reduction in PMD_20_ than HSD (Fig. S11 and S12). We also performed overnight plate-reader assay to determine the AgNP’s effects on the growth rates. Intriguingly, the plate-reader assay showed that HST and FU nanoparticles are more effective in inhibiting growth than HSD, which was consistent with PMD_20_ (Fig. 4C). Therefore, this result suggests that BeAST can be used for examining antifungal activities based on membrane potential response dynamics to electric stimulation.

## DISCUSSION

This study presents BeAST based on the finding the electrically induced dynamics of the plasma membrane potential correlates with the inhibition of growth rates. We observed a correlation between growth rates and hyperpolarization, suggesting that BeAST can estimate the magnitude of stress. Strikingly, this relationship appears to be independent from molecular modes of action of antimicrobials.

We show that an electric stimulation hyperpolarizes the plasma membrane. This conclusion is supported by the measurements with two membrane-potential indicators: anionic DiBAC4(3) and cationic ThT, which indicate that the result is not dependent on specific dyes. Furthermore, it suggests that these plasma membrane potential dynamics may be an evolutionary conserved feature, since the prokaryotic organisms *Bacillus subtilis* and *Escherichia coli*, and the eukaryotic model *Saccharomyces cerevisiae*, show similar membrane potential dynamics in response to an electrical shock (15). The exact mechanism by which an electric stimulation triggers such an event requires further investigation. For instance, it is not clear whether EEF directly or indirectly impacts cellular membrane and membrane proteins. An important next step for microbial electrophysiology is to characterize the mechanism and explore possible explanations for why such a feature may be conserved.

We believe that BeAST has many potential applications in both industrial and laboratory settings. When applied to cellular populations, such an assay can be used to determine viability, defined as the number of vital cells. *S. cerevisiae* is an organism with significant applicability in biotechnological processes, which range from brewing and baking to the production of pharmaceutical molecules and expression of heterologous proteins (29, 30). However, yeast can also be a concerning contaminant in industrial settings, with 9% of non-food product recalls being caused by yeast and mold (31). Thus, rapid assessment of vitality and viability to assure either efficient bioprocesses in food and biotechnology industries, or efficient elimination of contaminants, is a pressing need. The traditional methods for monitoring yeast viability rely on growth analysis methods including colony-forming units (CFU) and spotting tests, or on live/dead cell-staining methods including methylene blue and phloxine B (32). These methods, however, are not suited for measuring vitality. Vitality measurements generally rely on monitoring cellular growth or metabolic activities. While these methods are widely used and well established, conducting and analyzing them take several days or require complex calibrations. Flow cytometry is a sophisticated technology that can provide rapid results of live cell detection; however, it requires technical expertise and its ability to estimate the growth rate may be limited. Our work suggests that electrical stimulation can shorten the time it takes to do such an assay. Additionally, this approach could accelerate the screening of anti-fungal compounds and potential therapeutic agents for fungal infections. It could also be useful for basic research using *S. cerevisiae* where measuring cell growth rates is important. For example, the growth rate and electrophysiology properties of *S. cerevisiae* have been shown to correlate to aging (33, 34). A future study shall examine the relationships between PMD and CCCP concentrations more granularly and explore different types of stressors to quantitatively characterize the relationship between stress and PMD. It is yet to be seen if BeAST can be applied with clinically relevant fungal species and strains. If they do, estimating the growth inhibition levels by BeAST may shorten the time it takes for diagnosis and could uncover the roles that bioelectricity plays in a plethora of cellular processes. Thus, BeAST has a potential to aid the diagnosis of fungal infections and development of antifungals.

## MATERIALS AND METHODS

### Strain and growth condition

*Saccharomyces cerevisiae* (haploid, W303 background) was cultivated in yeast nitrogen base (YNB) (Foremedium) with Complete Supplement Mix (CSM) (Foremedium) liquid media supplemented with 2% glucose (wt/vol) and on yeast extract peptone dextrose (YEPD) agar (1.5% [wt/vol]). For time-lapse and electrical stimulation experiments, a single colony of *S. cerevisiae* was inoculated into YNB + 2% glucose media and cultured at 30°C in a shaking incubator overnight. In the following morning, cells were diluted into YNB + 2% glucose to OD_600_ = 0.15–0.2 and allowed it to grow at 30°C, shaking until reaching OD_600_ = 0.6. When specified so in the figure legend, carbonyl cyanide chlorophenylhydrazone (CCCP) was added to the liquid culture to the final concentration of 10, 40, or 100 µM in the last 1 h of incubation. Cells were then inoculated onto YNB 2% glucose supplemented with low-melting-point agarose (1.5% [wt/vol]) pads with membrane potential dye. Pads were prepared as described previously (35) by melting agarose (Fisher bioreagents—low melting point agarose) into YNB 2% glucose liquid media, then pouring 1 mL onto 22 mm × 22 mm glass slip and covering with another glass slip to it to solidify. Agarose was cut into 5 mm × 5 mm pads. Three to five microliters of culture was inoculated on individual pads that were then placed onto a willco dish for general time-lapse experiments or placed onto a gold-coated glass-bottom dish for electrical stimulation experiments (15, 16).

### Time-lapse microscopy

Yeast membrane potential dynamics was observed using a Nikon TI-E Eclipse motorized wide-field epifluorescence microscope equipped with an EM-CCD camera (Andor DU-888). Operation of the microscope was performed using NIS Elements (Nikon) and temperature control was obtained by an incubation chamber (Okolab Bold Line Cage Incubator). Before time-lapse experiments, chamber temperature was set at 30°C for at least an hour; then samples were placed into the chamber a further hour before starting the experiment. Time-lapse experiments were performed using 100× objective lens (N.A. 1.45, Nikon). Cell growth was recorded using phase contrast. Membrane potential dynamics was monitored by the combination of the excitation laser and emission filter adequate for the dye used in that specific experiment. ThT fluorescence was detected using a 405 nm excitation laser and a QUAD emission filter. TMRM fluorescence was detected using a 561 nm excitation laser and a QUAD emission filter. DiBAC fluorescence was detected using a 488 nm excitation laser and a GFP-FITC (525/50) emission filter.

### Electrical stimulation experiments

Electrical stimulation experiments were performed as described previously (15, 16). Briefly, stimulation was performed by applying an alternating current (AC) signal (0.1 kHz; 3 V peak-to-peak [−1.5 ∼+1.5 V]) to individual electrodes of the gold-coated glass-bottom dish using CytePulse (Cytecom Ltd). Cell growth was monitored prior to electrical stimulation using an inverted epifluorescence microscope, DMi8 (Leica Microsystems), operated by MetaMorph (Molecular Devices) and equipped with a scientific CMOS camera ORCA-Flash 4.0 v2 (Hamamatsu Photonics). All experiments were conducted at 30°C, maintained by an i8 incubation chamber (Pecon). Samples were placed into the chamber for 1–2 h for thermal equilibration. Observations were performed using a 100 × objective lens (N.A. 1.3, HCX PL FLUOTAR; Leica). Growth was observed with bright field and membrane potential dynamics was monitored using ThT, TMRM, or DiBAC4(3). ThT fluorescence was detected using a 438/24 nm excitation filter, a 483/32 emission filter, and a 458 dichroic mirror (Semrock). TMRM was detected using a 554/23 excitation filter, a 609/54 emission filter, and a 573 dichroic mirror (Semrock). DiBAC4(3) was detected using a 500/20 excitation filter and a 578/105 emission filter. Prior to electrical stimulation, cell growth was recorded for 1 h. For UV light exposure experiments, cells were exposed to UV-violet (UV-V) light for 1 min, using the DMi8 inverted microscope (Leica Microsystems) and the LED light source, SOLA SM II Light Engine (Lumencor) equipped with an excitation filter of 400/16 nm. The area exposed to UV light was controlled by the field diaphragm of the microscope.

### Biogenic silver nanoparticles

Biogenic silver nanoparticles were prepared using the protocol described by Stanisic et al. (27), using flavonoids from oranges—hesperetin (HST) and hesperedin (HSD)—as reducing and stabilizing agents. Hesperidin (HSD) and its aglycone, hesperetin (HST), are two flavonoids from citrus species that have various biological properties and take part in a biogenic silver nanoparticle (AgNP) synthesis in the same way, using flavanone moiety in an oxidation/reduction reaction. Along the synthesis, HSD and HST stabilize the AgNPs by interaction with the AgNP surfaces. Therefore, it was expected that AgNP-HSD and AgNP-HST would show very similar properties and activities. The only structural difference is the disaccharide rutinose moiety in HSD as illustrated below. The synthesized AgNP-HSD and AgNP-HST were spherical in shape (26), exhibited low zeta potentials (around −35 meV), polydispersity around 0.4, and 25–30 nm in diameters. The unique difference between them was the sugar moiety (O-rutinoside) on HSD. We have fully characterized AgNPs using dynamic light scattering, surface charge measurements, and transmission or cryogenic electron microscopy. See also the supplemental information. Fungal biogenic silver nanoparticles (AgNP-FU) were prepared according to the protocol described by Ballotin et al. (33, 36) using the fungal filtrate prepared from the Fusarium oxysporum biomass. The synthesized AgNP-FU were spherical, exhibited low zeta potential (−34.5 ± 5.4 mV), acceptable polydispersity, and 25 nm in diameter. Using dynamic light scattering, surface charge measurements, and cryogenic transmission electronic microscopy, AgNP-FU particles were fully characterized as shown in the supplemental material.

### FttzHugh-Nagumo model

The electrically induced membrane potential dynamics was simulated using the FitzHugh-Nagumo (FHN) neuron model, similar to a previous work (15). The membrane potential *Vm* and the recovery variable *W* were considered by the following equations:


$$\frac{dV_{m}}{dt}=k_{K}\left(V_{m}-V_{m}^{3}+W\right)+\frac{I_{v}}{dt}\;\frac{dW}{dt}=\;-V_{m}+\beta -W+\frac{dI_{w}}{dt}$$

where $I_{v,w}$ is the externally applied electrical field (EEF) and *k_K_* represents cell vitality. *b* was defined as:


$$\beta =\frac{1-0.1\log k_{K}}{2}$$

The equations were solved in Python using a scipy ode solver (scipy.integrate.odeint). EEF was applied to the equilibrium state. To analyze the impact of cellular states, the simulations were conducted for $k_{K}$ between 0.1 and 1, and the ratio changes in *V* were recorded. The fluorescence intensity (*I*) was then calculated as ${I=e}^{\alpha V}$ . The ratio change in *I* was plotted. The growth rate was assumed to be linearly proportional to *k_K_*.

## AFTER EPUB

*[This article was published on 23 July 2024 with an error in the introduction. The text was corrected in the current version, posted on 26 July 2024.]*

## SUPPLEMENTAL MATERIAL

Supplemental material mbio.01302-24-s0001.pdf Figures S1 to S15, Table S1, and supplemental methods.

Movie S1 mbio.01302-24-s0002.mp4 Proliferative cells stained with DiBAC4(3) stimulated by EFF, corresponding to Fig. 2B.

Movie S2 mbio.01302-24-s0003.mp4 Proliferative cells stained with ThT stimulated by EFF, corresponding to Fig. 2C.

Movie S3 mbio.01302-24-s0004.mp4 Proliferative cells stained with TMRM stimulated by EFF, corresponding to Fig. 2D.

Movie S4 mbio.01302-24-s0005.mp4 Yeast cells stained with ThT, exposed to UV, corresponding to either Fig. 3B or Fig. 3D.

ASM does not own the copyrights to Supplemental Material that may be linked to, or accessed through, an article. The authors have granted ASM a non-exclusive, world-wide license to publish the Supplemental Material files. Please contact the corresponding author directly for reuse.
